# Validation of an on-chip p16^ink4a^/Ki-67 dual immunostaining cervical cytology system using microfluidic device technology

**DOI:** 10.1038/s41598-023-44273-6

**Published:** 2023-10-10

**Authors:** Kei Hashimoto, Tomoo Kumagai, Kyosuke Nomura, Yuko Miyagawa, Saori Tago, Kazuki Takasaki, Yuko Takahashi, Haruka Nishida, Takayuki Ichinose, Mana Hirano, Haruko Hiraike, Osamu Wada-Hiraike, Yuko Sasajima, Soo Hyeon Kim, Kazunori Nagasaka

**Affiliations:** 1 Department of Obstetrics and Gynecology, Teikyo University School of Medicine, Kaga 2-11-1, Itabashi-Ku, Tokyo, 173-8605 Japan; 2 Institute of Industrial Science, University of Tokyo, Tokyo, Japan; 3 Department of Obstetrics and Gynecology, Graduate School of Medicine, The University of Tokyo, Tokyo, Japan; 4 Department of Pathology, Teikyo University School of Medicine, Tokyo, Japan

**Keywords:** Gynaecological cancer, Biotechnology, Cancer, Biomarkers, Biomedical engineering

## Abstract

More specific screening systems for cervical cancer may become necessary as the human papillomavirus (HPV) vaccine becomes more widespread. Although p16/Ki-67 dual-staining cytology has several advantages, it requires advanced diagnostic skills. Here, we developed an automated on-chip immunostaining method using a microfluidic device. An electroactive microwell array (EMA) microfluidic device with patterned thin-film electrodes at the bottom of each microwell was used for single-cell capture by dielectrophoresis. Immunostaining and dual staining for p16/Ki-67 were performed on diagnosed liquid cytology samples using the EMA device. The numbers of p16/Ki-67 dual-stained cells captured by the EMA device were determined and compared among the cervical intraepithelial neoplasia (CIN) lesion samples. Seven normal, fifteen CIN grade 3, and seven CIN grade 2 samples were examined. The percentage of dual-positive cells was 18.6% in the CIN grade 2 samples and 23.6% in the CIN grade 3 samples. The percentages of dual-positive staining increased significantly as the severity of the cervical lesions increased. p16/Ki67 dual immunostaining using the EMA device is as sensitive as the conventional method of confirming the histopathological diagnosis of cervical samples. This system enables a quantified parallel analysis at the individual cell level.

## Introduction

Cervical cancer is the fourth most common cancer in women worldwide in both incidence and morbidity^1^. Although human papillomavirus (HPV) is considered the cause of all histologic types of cervical cancer, some cervical cancers are not associated with an HPV infection. While over 90% of cervical cancers are caused by HPV infection^2^, most HPV infections are transient. When an HPV infection progress to cervical intraepithelial dysplasia (CIN), most CIN grade 1 (CIN1) and CIN grade 2 (CIN2) infections are transient^3^. The use of cervical cancer screening has decreased the incidence and mortality of cervical cancer since the introduction of the Papanicolaou test in the 1950s^4^, though cervical cancer remains a major health problem affecting women, especially those under the age of 40^5^. In Japan, approximately 10,000 people are diagnosed with cervical cancer annually and approximately 3000 die from this disease^6^. In Japan, cervical cancer screening is shifting from methods based on cytological diagnoses to a screening program that introduces HPV testing, and cervical cancer screening with HPV testing is expected to become more widespread^6^. Recent studies suggest that the combination of HPV testing with primary screening for cervical cancer allows the screening interval to be extended from five years to ten years, especially for women who are negative for high-risk HPV that causes cervical cancer^7–10^. Evaluations of several simulation models have shown that HPV screening reduces the overall incidence of cervical cancer^11,12^. However, long-term follow-up data and cost effectiveness analyses are needed, and errors of the age-group assessments and various HPV tests must be considered^13–15^. A new program in the Netherlands has extended the screening interval to ten years for women over 40 years of age^16^. However, patients with symptoms of mild irregular genital bleeding and abdominal pain, which occur during the early stages of cervical cancer, may no longer seek a cytological diagnosis if they received a negative HPV test within ten years of their symptoms.

In contrast, these patients would have undergone intermittent cytological diagnoses based on previous guidelines^17^. Therefore, in Japan, the transition of cervical cancer screening from a cytological diagnosis to a system in which HPV screening alone is the primary screening is controversial because of the quality of cytological diagnostic technology available^6^. In addition, the identification of an HPV infection during screening is not predictive of a patient’s prognosis, and the patient may not present to the hospital for triage after a positive HPV screening result^18,19^. However, if every patient who screened positive for HPV presented to the hospital, it may overwhelm the triage system. In addition, as HPV vaccination becomes more widespread, specimens with a cytological diagnosis of atypical squamous cells of undetermined significance (ASC-US) that are negative for HPV may be identified, and an auxiliary diagnostic method will be necessary^20–22^. Colposcopy and visual inspection with acetic acid may be used as auxiliary diagnostic methods. However, these diagnoses require a high level of skill, and more straightforward diagnostic methods need to be established. Additionally, cytodiagnostics and pathodiagnostics, especially in developing countries with high cervical cancer morbidity and mortality rates, are inadequate for medical education and systems^20^. Cytological diagnostic methods require multiple highly skilled cytologists to be involved in the diagnostic work, and standardization of cytological diagnostic techniques and diagnostic criteria, as well as accuracy control based on this standardization, are indispensable^17^. Therefore, the usefulness of the p16 and Ki67 dual-staining method of cytological diagnosis specimens, which has recently been reported as effective^23–26^, for triaging specimens that are not identified using HPV testing and those that are positive for HPV must be evaluated. The use of dual-staining methods can provide a newer and more objective perspective than cytological diagnostic methods, similar to the addition of the HPV test. However, the existing p16/Ki67 staining protocol requires technicians to possess advanced skills and the knowledge of cytology and several complex cell processing steps, such as attaching cells to slides. Therefore, new equipment with simpler and less labor-intensive cytology procedures are required^27–29^. This type of analysis may be possible using microfluidic technology.

Clinical devices using microfluidic technology have been developed for several applications^30,31^. However, this technology has not yet been applied to cytological diagnostic methods; this study is the first application of microfluidic technology for cytological diagnostic methods. Microfluidic technology allows for the analysis of cells at the single-cell level^32–34^, which has been verified using cultured cervical cancer cells^33^. However, cervical cancer cells, unlike cultured cells, are heterogeneous in size and shape. In addition, specimens stored in liquid cytology also contain non-cellular impurities including blood, mucus, and bacteria, which must be removed as much as possible prior to processing. In this study, the microfluidics device was improved, and the automated diagnosis of cytology specimens fixed by on-chip liquid cytology was verified.

## Results

### Creation of devices for more effective cervical cell capture and immunostaining analysis

**Figure 1:**
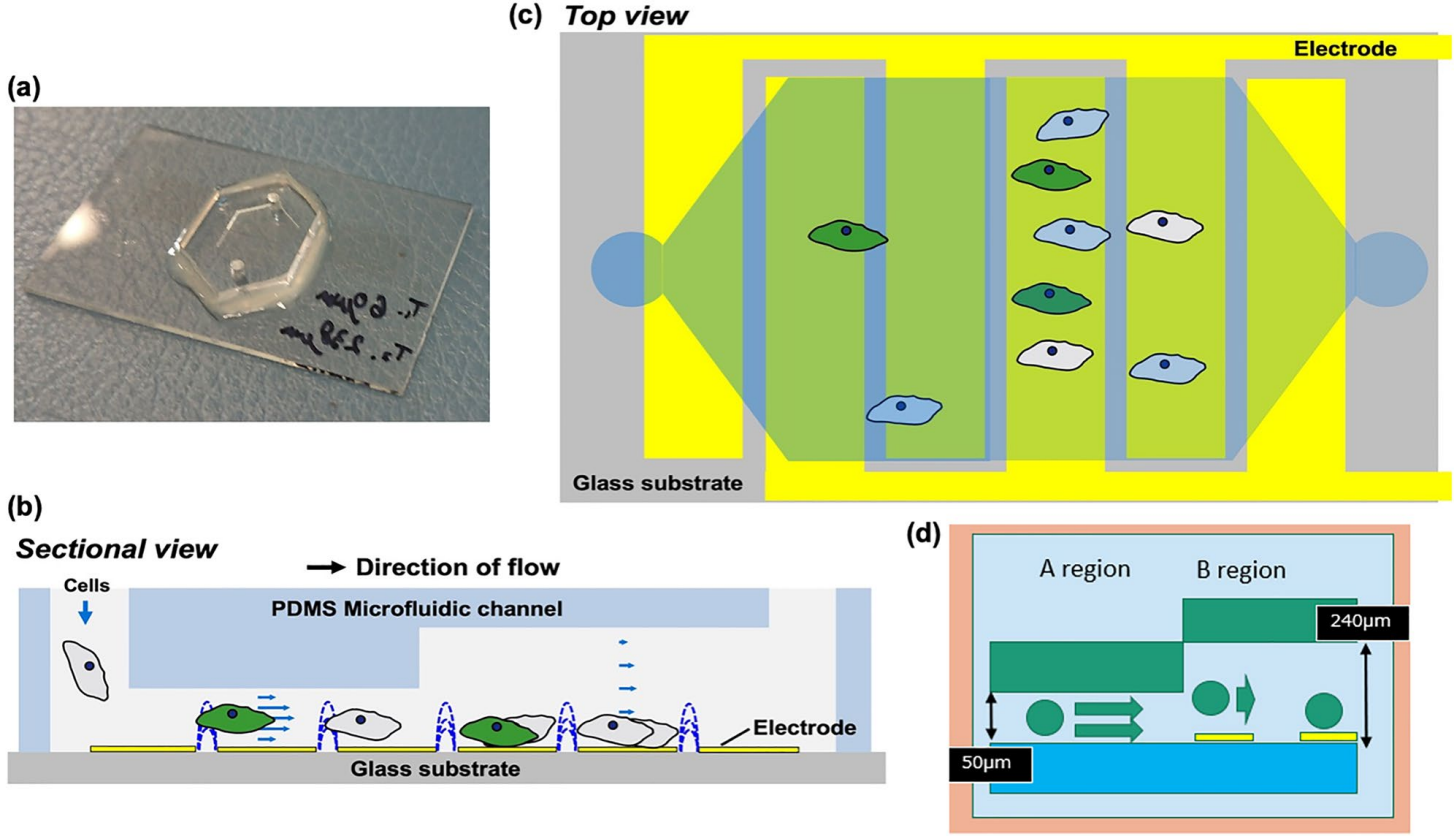
Design of the electroactive microwell array (EMA) STEP device. (**a**) The microfluidic device (EMA STEP) used in the study. (**b**) Sectional view of the microfluidic device. (**c**) Patterned interdigitated electrodes are aligned on a glass substrate, consisting of an A region and a taller B region. No microwells are included. The difference in height creates a change in flow velocity as samples transition from region A to region B. (**d**) The change in flow velocity makes cell capture easier. The surface of the flow channel is coated with an anti-adsorption material.

**Figure 2:**
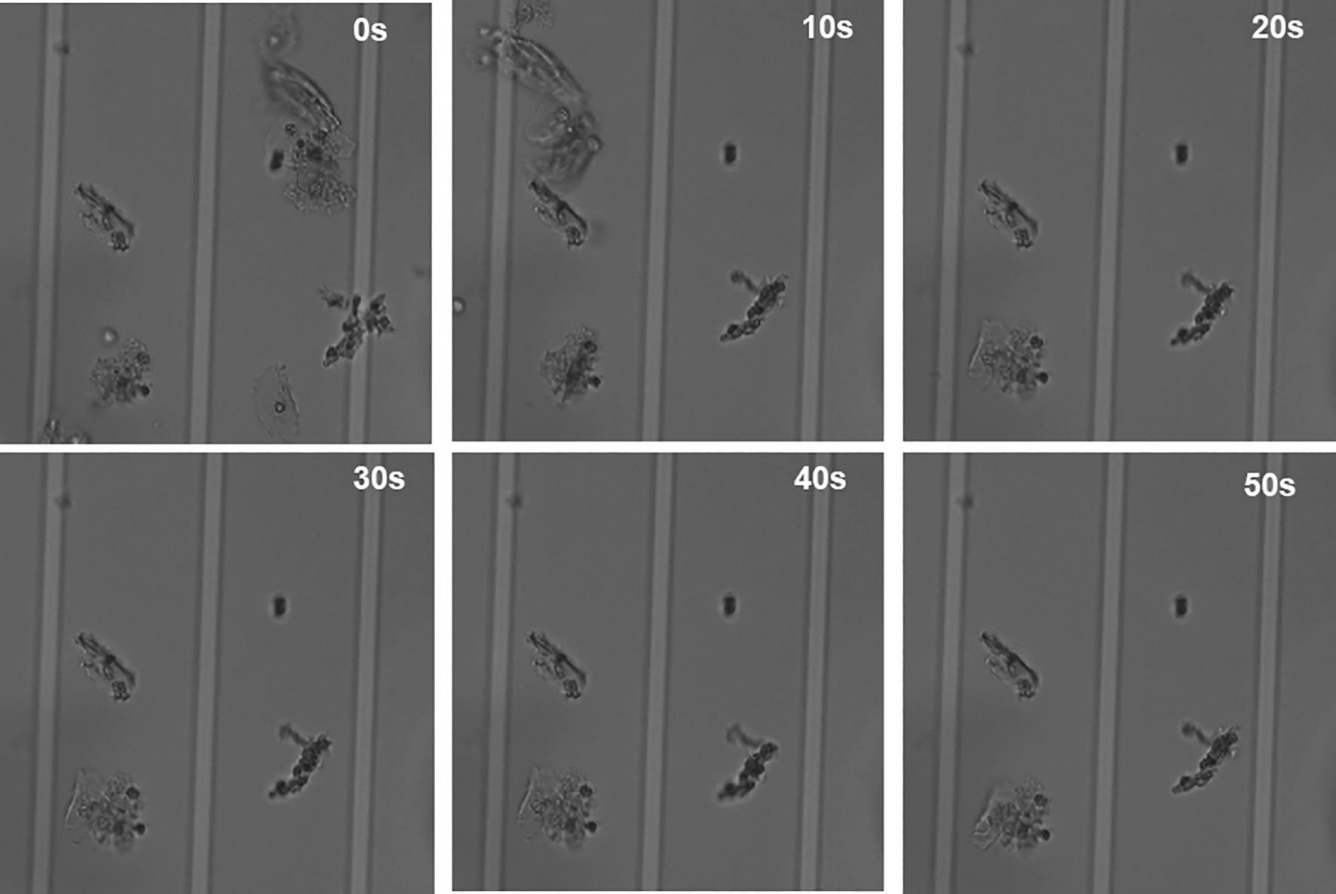
Sample cells trapped by the EMA STEP device in brightfield. Time-lapse images captured from the start of the dielectrophoresis are shown (0, 10, 20, 30, 40, and 50 s). There are cells flowing from the right to the left in the device, but some cells are captured and retained between the electrodes at 0, 20, 30, 40, and 50 s. Single cells are trapped by negative dielectrophoresis (nDEP).

Uterine cervical cell samples contain cervical-derived cells, blood, mucus, and bacteria. Therefore, the cells were fixed using an existing liquid mace cytology kit. The fixed cells are stable for approximately two weeks. To capture and analyze fixed cells of various shapes, the device could not include microwells, as shown in Fig. 1, as some cells would not be captured when microwells were placed owing to the variations in cell size. Therefore, a device with steps was designed (Fig. 1) and DEP was used to separate cells with different shapes. On time-lapsed, bright-field imaging of cervical cells in the device, some cells seem clumped together, though most cells are singular (Fig. 2). The cells are trapped on the electrode using DEP.

### Cells floating on a liquefied basis need to be analyzed in three dimensions

**Figure 3:**
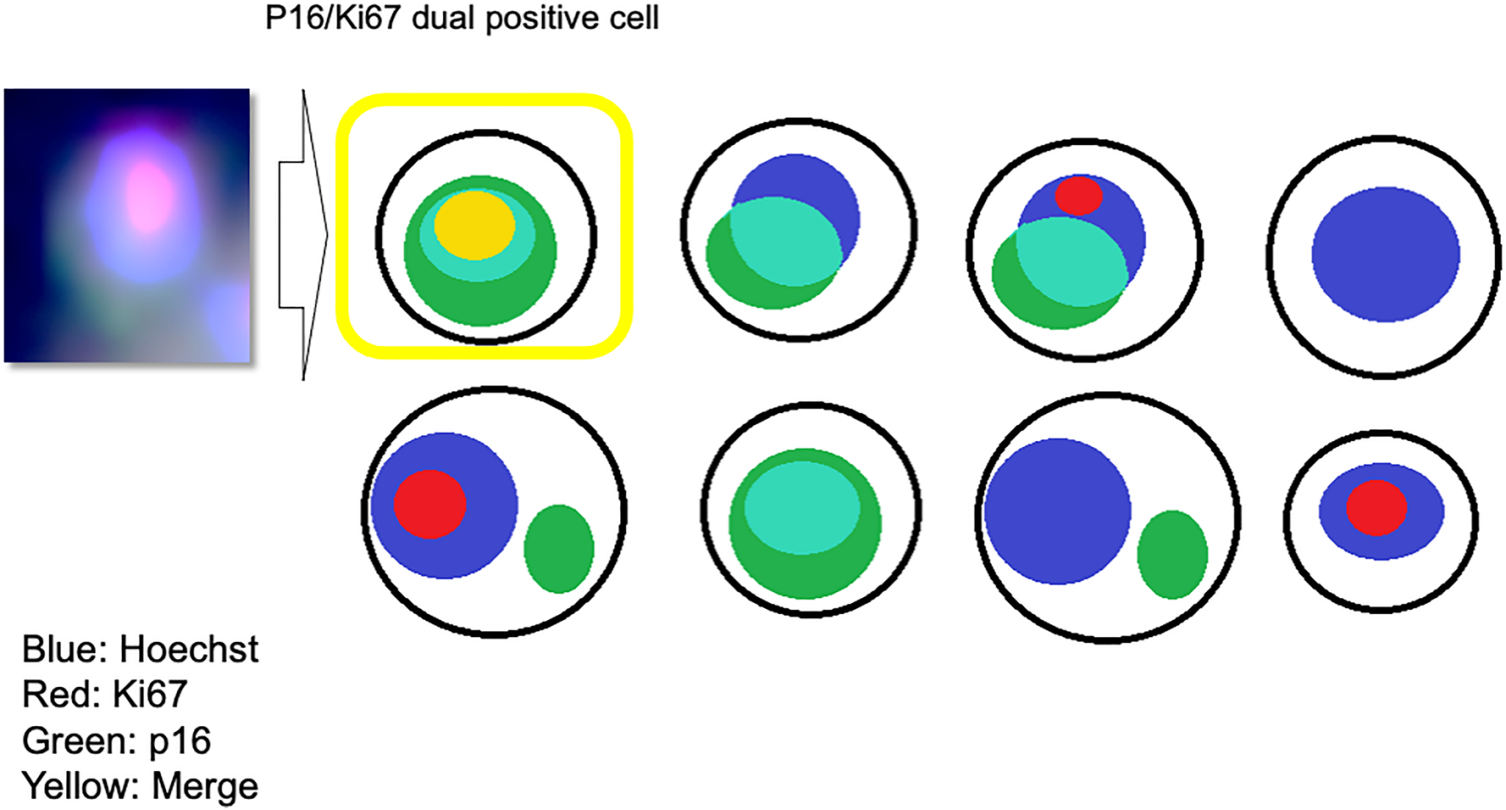
Positive cells. When analyzing fluorescent staining, it is not easy to count cells with multiple fluorescent colors. Therefore, based on the ease of measurement and the three-dimensional structure of the cells, positivity is defined as the point where the Hoechst (blue), Ki67 (red), and p16 (green) stains merged in the same spot. The ratio of positive cells to total cells is determined instead of the absolute number of cells. Randomness due to the unadjusted number of cells and irregular rotation of cells due to the flow is unlikely to affect the ratio.

In this study, immunostaining was used to analyze cervical cells captured within the device. While stratified cells on a slide can be observed in two dimensions if the cells are mounted so that they do not overlap, as in case of the cytology procedures, the suspended cells can be observed in three dimensions. A great deal of information can be gained by observing cells in three dimensions in terms of the overlap and localization of stained areas within the cell. The device has an inlet for injecting antibodies and buffer and an outlet used for draining (Fig. 1). Therefore, the cells suspended in the device are captured when the dielectric flow is applied. During routine pathological examinations, cells are attached to a slide and Papanicolaou staining or immunostaining is performed on the cells in two dimensions. Immunostaining is often used for histological diagnoses and is rarely used for cytological diagnoses as this technique is not well established for the latter. Analysis using a laser microscope is needed, especially when fluorescent antibodies are used. In this study, cells were stained with fluorescent p16 and Ki67 antibodies. It was difficult to automatically count cells with multiple fluorescent colors to define the positive cells that were stained with Hoechst (blue), Ki67 (red), and p16 (green) (Fig. 3). The ratio of positive cells to the total cells was determined. As the number of cells was not adjusted prior to the measurement and irregular rotation caused by specimen flow resulted in randomly-arranged cells, these factors were unlikely to affect the positive cell ratio.

### Immunostaining of cervix-derived cells fixed via liquid-based cytology using the device

**Figure 4:**
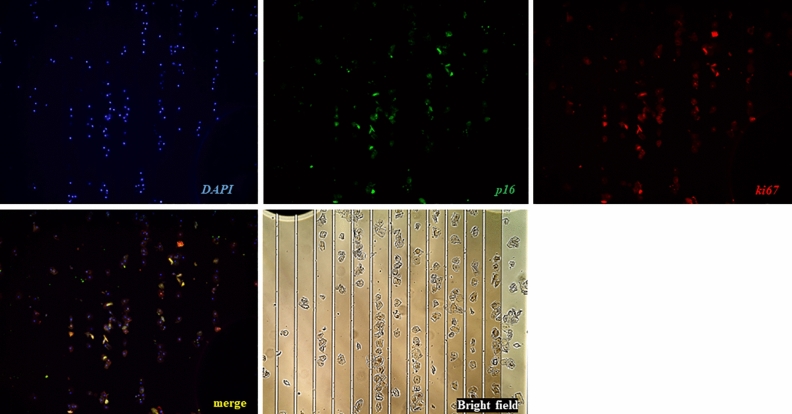
Immunostaining assay of specimens trapped by the EMA STEP device. Cervical cells obtained from patients were successfully captured and stained with the target proteins Ki67 and p16 and evaluated using the microfluidic device. Cells showing dual staining with p16 and Ki67 can be visualized in yellow on Merge for further assessment.

**Figure 5:**
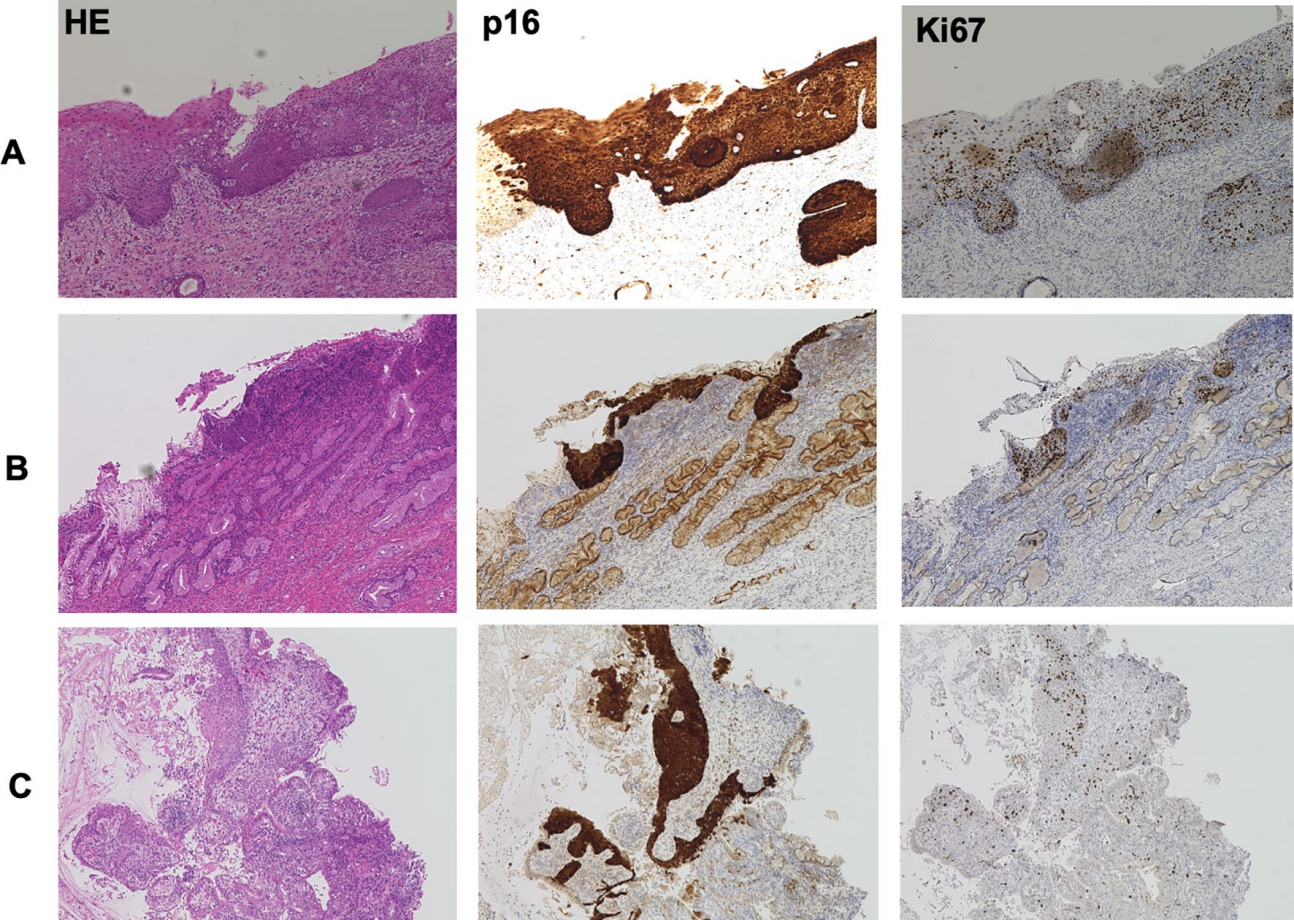
A p16/Ki67 dual immunostaining in histopathologic diagnosis. (**a**) Cervical intraepithelial neoplasia grade 3 (CIN3) cervical tissue (Case # 27). (**b**) CIN3 cervical tissue (Case # 23). (**c**) Cervical intraepithelial neoplasia grade 2 (CIN2) cervical tissue (Case # 13). Continuous and diffuse p16 and Ki67 staining from the basal/parabasal layers to the middle/superficial layers is shown, indicating overexpression. Automated analysis of histologically diagnosed samples were analyzed in the study. *HE* Hematoxylin–Eosin.

The cervix-derived cells injected into the device and captured on the electrode could be modulated by switching the electrode on and off (Fig. 4). These cells were obtained from a patient diagnosed with CIN grade 3 (CIN3). Hoechst (blue), Ki67 (red), and p16 (green) immunostaining was performed, and cells positive for both Ki67 and p16 were observed (Fig. 4). Examples of the immunohistochemical staining of three cervical cell specimens are shown in Fig. 5. The rate of p16 and Ki67 staining in the CIN2 and CIN3 specimens increased as the disease severity progressed.

### p16/Ki67 staining using the on-chip microfluidic device can accurately assess the disease status

**Figure 6:**
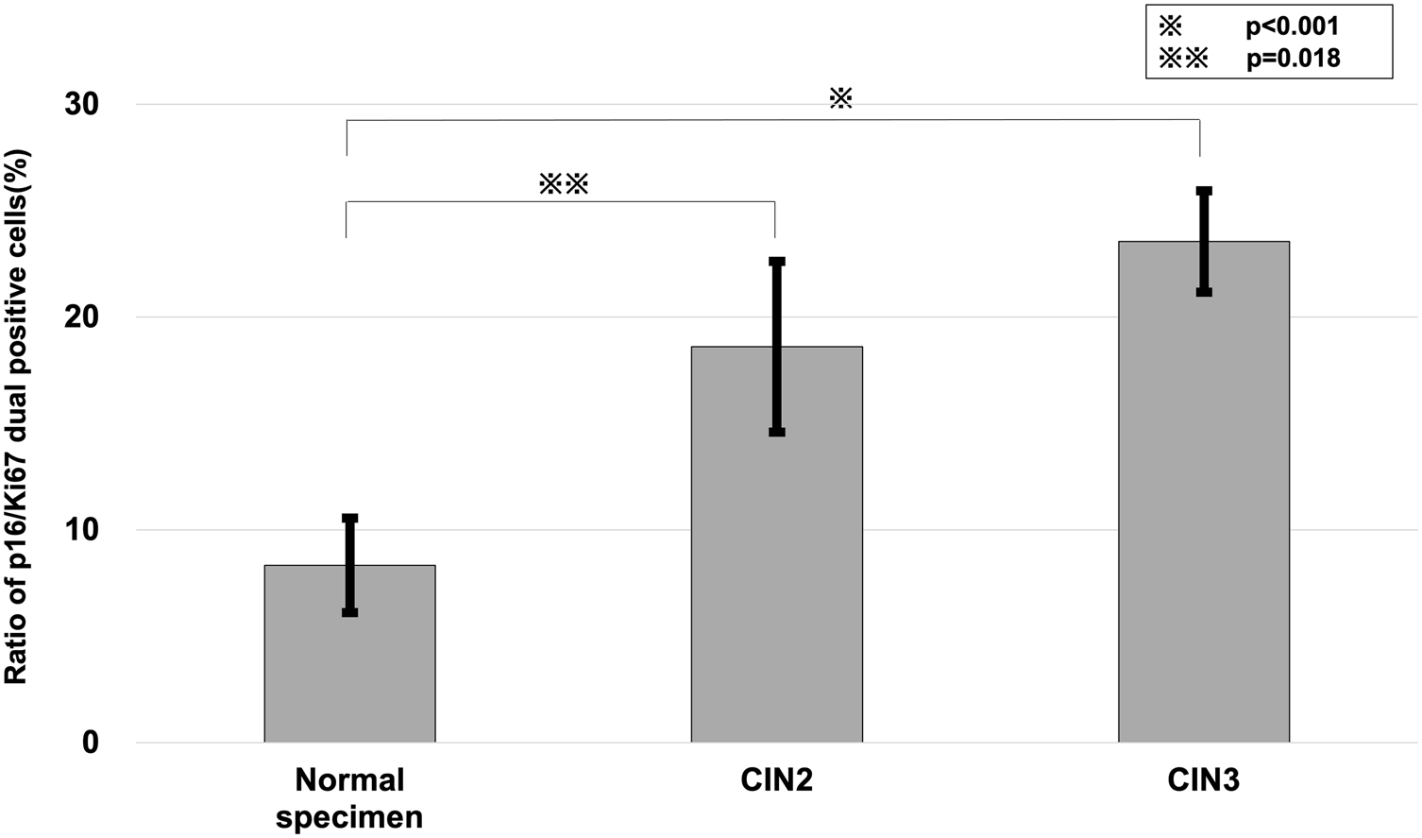
Relationship between cervical lesions and positive cell ratio. Significant differences in the positive ratio were found according to the severity of the cervical lesions. The degree of p16 and Ki67 dual-staining positivity increased as the disease severity increased (Normal specimen vs CIN2: p = 0.018, Normal specimen vs CIN3: p < 0.001, as assessed by Mann–Whitney *U* tests). *Normal specimen* normal cell cytology, *CIN2* cervical intraepithelial neoplasia grade 2, *CIN3* cervical intraepithelial neoplasia grade 3.

The 29 cervical cell specimens used in this study were stored in liquid-based cytology vials and analyzed within two weeks of cell fixation. Concurrent pathological diagnoses were made via conventional cytology and blinded to the researchers. In Japan, HPV testing is unavailable by medical insurance unless the patient is diagnosed with ASC-US or CIN2. Therefore, HPV typing was performed on specimens for which HPV testing was available (Table 1). p16 and Ki67 dual-staining positivity increased as the disease severity increased (Normal specimen vs CIN2: p = 0.018, Normal specimen vs CIN3: p < 0.001 by Mann–Whitney U tests) (Fig. 6). Pre-processing of the specimen resulted in the separation of cells to a single cell or a few cell clusters, enabling the evaluation of the positive cell rate. There are two possible reasons for the p-value of 0.018 in the differentiation between the normal and CIN2 specimens. One is the possibility that atypical cells positive for p16 and Ki67 were included in the cytodiagnostically normal specimens. The other is the presence of near normal pathology in the cases pathologically diagnosed as CIN2. However, CIN3 is considered less likely to be missed, and the evaluation of atypical cells by microfluidic devices accurately diagnosed CIN3 specimens (Fig. 6).

**Table 1:** Clinicopathological background of clinical specimens (n = 29).

Sample no.	Cytology	Histological diagnosis	HPV genotype	Ratio of dual positive cells (%)
1	NILM	Negative	N/A	10.3
2	NILM	Negative	N/A	5.9
3	NILM	N/A	N/A	10.0
4	NILM	N/A	N/A	11.1
5	NILM	N/A	N/A	8.3
6	NILM	N/A	N/A	5.6
7	NILM	N/A	16 +	6.3
8	HSIL	CIN2	52 +, 58 +	31.7
9	HSIL	CIN2	Other +	18.6
10	ASC-US	CIN2	31 +, 52 +, 56 +	27.9
11	HSIL	CIN2	52 +	33.8
12	HSIL	CIN2	16 +	8.1
13	ASC-H	CIN2	52 +	10.1
14	HSIL	CIN2	Positive (type not determined)	13.2
15	HSIL	CIN3	18 +, 52 +	34.2
16	HSIL	CIN3	16 +	16.9
17	HSIL	CIN3	18 +	25.0
18	HSIL	CIN3	16 +	20.0
19	HSIL	CIN3	Positive (type not determined)	10.7
20	HSIL	CIN3	16 +	27.5
21	HSIL	CIN3	16 +, 52 +, 56 +, 58 +	15.2
22	HSIL	CIN3	Positive (type not determined)	23.6
23	HSIL	CIN3	35 +, 51 +, 58 +, 59 +	22.2
24	HSIL	CIN3	52 +	33.9
25	HSIL	CIN3	52 +	36.4
26	ASC-US	CIN3	52 +	8.3
27	HSIL	CIN3	16 +, 52 +	30.0
28	HSIL	CIN3	52 +, 58 +	13.7
29	HSIL	CIN3	16 +	34.5

*NILM* Negative for Intraepithelial Lesion or Malignancy, *HSIL* High-grade Squamous Intraepithelial Lesion, *ASC-US* Atypical Squamous Cells of Undetermined Significance, *ASC-H* Atypical Squamous Cells, cannot exclude High-grade squamous intraepithelial lesion, *CIN2* cervical intraepithelial neoplasia grade 2, *CIN3* cervical intraepithelial neoplasia grade 3, *N/A* not available.

## Discussion

Research regarding microfluidic devices has recently improved^30,33,34^. Microfluidic devices are fabricated using semiconductor microfabrication technology and precision machining technology^35^ to form microscopic flow paths on substrates such as resin and glass to freely mix and distribute liquids and fine particles flowing in liquids on a micro-scale^36^. The technology is expected to become a chip-type functional component with a wide range of applications in drug discovery, healthcare, microchemical synthesis, and genetic analyses^37^. Microfluidic devices include organ-on-a-chip (OOC)^38^, which reconstructs organ models on microfluidic devices; body-on-a-chip (BOC)^39^, which models individual animal responses; and lab-on-a-chip (LOC)^29^, which uses DEP. The microfluidic device used in this study is based on DEP, which has been studied as an adjunct test that can be used for secondary cervical cancer screening^40^. In a previous study, immunostaining was possible within the EMA device^33^. However, previous studies used clinical specimens derived from the actual cervix. As cervix-derived cells vary in shape and size, the development of a device without microwells was necessary^33^. Although an electroactive microwell array with barriers (EMAB) device^33^ was developed, because cells of cervical origin often form clumps, staining cannot be performed efficiently given that it is quite difficult to stain the entire clump of cells. However, with sufficient permeabilization and sonification, the cells were separated to the single-cell level (Fig. 3). After preliminary experiments were conducted, the clinical specimens were evaluated using the microfluidics device.

Primary screening for cervical cancer using HPV testing, which is currently used in Western countries, requires triaging to determine which patients who are positive for HPV should be referred for further testing^41,42^. Cytology diagnostic methods, which have been used for primary screening worldwide, can be used for triaging patients, p16/Ki-67 dual-stain cytology analyses, and testing HPVE6/E7 mRNAs, which are proposed as biomarkers for cervical cancer^43–45^. The p16/Ki-67 dual-stain cytology method can be used to stratify patients based on their risks and is useful to differentiate between ASC-US and low-grade squamous intraepithelial lesions^46^. Dual-stain cytology has also been reported to be suitable for triaging specimens with positive HPV tests and negative cytology^47,48^. Furthermore, the combination of cytologic diagnosis with p16 immunostaining and HPV typing for types 16 and 18 is as accurate as a colposcopy^49^. p16 staining alone can identify HPV infections and may enable the detection of abnormal cells. However, dual staining for p16 and Ki67 enables the detection of the proliferative capacity and the genomic instability of atypical abnormal cells^50^. In addition, HPV testing is used to triage patients with a cytological diagnosis of ASC-US, and p16/Ki-67 dual staining is significantly more specific than p16 staining alone^51^. As shown in a previous study^33^, flow-through immunostaining on the device is possible for the HeLa cell line, a commercially available cervical cancer cell line. Based on the results of the present study, we believe that a similar technique can be introduced for patient specimens; we plan to introduce this technique. Thus, we believe that the number of steps required by the user in the staining process can be reduced. Although some equipment is required because the samples used are liquid samples, once a more sophisticated method is established, the tests can be performed simultaneously and multiple times, and the benefits will outweigh the time and effort required.

This study has several limitations. First, the number of samples was small, and the collection of normal (control) pathological specimens was difficult as the specimens were not evaluated prospectively. Furthermore, the samples were collected with the patients' consent during routine healthcare screening. In this limited research environment, the accuracy of a microfluidic device for the diagnosis of cervical abnormalities was evaluated. The described method is intended for the development of an automated diagnostic instrument as an auxiliary diagnosis, not as a method for primary screening. If an instrument using a microfluidic device is developed, it may be possible to identify a single cell suspected to indicate CIN3- or a high-grade atypical lesion, even with a normal cytological diagnosis and negative HPV result. These preliminary results will continue to be analyzed in a prospective, large-scale, clinical study.

## Conclusions

The present study describes the development of a method to capture cervix-derived cells of different shapes and sizes at the single-cell level or as cell clusters using DEP in a stepped microfluidic device in which immunostaining can be performed. p16 and Ki67 dual staining has been applied clinically as a primary screening method and a precision examination and may soon be accepted as a diagnostic method for cervical lesions. The automation of this method requires advanced diagnostic techniques.

Fluorescent antibodies were used in this study; this enabled the evaluation of the stained cells using a laser microscope. The development of a system that enables the detection of positive cells using only a laser should be considered. These developments will make cervical cancer screening more accurate and accessible worldwide.

## Methods

### Device fabrication

The microfluidic device consists of a glass substrate containing an indium tin oxide (ITO) electrode and a polydimethylsiloxane (PDMS) fluidic channel. First, electrode patterns were designed using computer-aided design software (LayoutEditor, Juspertor GmbH, Unterhaching, Germany), and the ITO electrodes were fabricated by GEOMATEC Co., Ltd. (Tokyo, Japan). A PDMS step-channel was fabricated using a standard replica molding process^33^. A negative-type photoresist (SU-8 3035, MicroChem Co., MA, USA) was spread onto a silicon wafer to create a layer of the channel structure with a thickness of 50 µm. The wafer was then soft baked at 95 °C for 15 min, and a chromium photo-mask patterned for the thin fluidic channel was placed in contact with the wafer and exposed to ultraviolet light. After exposure, the wafer was baked at 95 °C for 5 min, followed by development and rinsing using the SU-8 developer and isopropyl alcohol, respectively. An additional thick channel structure was fabricated on the thin mold substrate to create a step. The thin mold substrate was coated with a negative-type photoresist (SU-8 2100). The substrate was then soft baked, and a chromium photo-mask patterned for the thick channel was aligned with the thin mold and exposed to ultraviolet light. After exposure, the substrate was baked at 95 °C and developed and rinsed using SU-8 developer and isopropyl alcohol, respectively. A prepolymer of PDMS was mixed with a curing reagent (10:1 mass ratio) and poured over the mold master. Then, the PDMS was cured at 75 °C for 2 h. After curing, the polymerized PDMS layer was removed from the mold. Holes that served as access ports to the flow channels were punched out using a 1-mm biopsy punch. The PDMS channel and ITO electrode substrate were exposed to O_2_ plasma using a reactive ion etching machine (RIE-10NR, Samco Co., Kyoto, Japan), and placed in contact with the device for permanent bonding. The microfluidic device was placed on an X–Y translation stage on an all-in-one fluorescence microscope (BZ-X800; Olympus, Tokyo, Japan). A camera that was built into the microscope system was used to monitor the cells within the device, and the potential for dielectrophoresis (DEP) trapping was applied to the ITO electrode using a function generator (WF1948; NF Corporation, Yokohama, Japan). The outlet of the microfluidic device was connected to a gas-tight glass syringe (Hamilton Company, NV, USA) The syringe was operated using a precision-controlled syringe pump (YSP-201; YSP-201, Kyoto, Japan).

### Patients and specimens

Cervical cell specimens were collected from April 2020 to April 2022 by a physician at Teikyo University Hospital (Table 1). A total of seven CIN2, fifteen CIN3, and seven normal specimens were used in this study. The diagnosis of each specimen was confirmed via cytology and histology analyses. After collection, the cervical cell samples were fixed as liquefied specimens using ThinPrep^Ⓡ^ (Hologic Inc, Bedford, MA, USA). All patients provided written informed consent for the research use of their samples, and the collection and use of tissue for this study was approved by the Clinical Ethics Committee of the Medical Faculty at Teikyo University (approval number: 13-003-4 on 22 October 2020). This study was conducted in accordance with the Declaration of Helsinki. All samples were blinded and clinically evaluated by three independent pathologists as a part of the routine pathological diagnosis.

### Immunostaining

To make the staining and trapping process more effective, 3 mL (cell count: 1 × 10^5^/mL–1 × 10^6^/mL) of the liquid sample was collected; this was sufficiently suspended to avoid sedimentation, and the liquid specimens were filtered through a 100-μm filter to remove the cell clumps. In addition, ultrasonic vibration was applied at a frequency of 50 kHz for 3 min to separate the cell clumps. Immunostaining of the cervical cells targeting the Ki-67 and p16 proteins was performed using the CINtecPLUS immunostaining protocols (Roche MTM Laboratories, Heidelberg, Germany). The cells were permeabilized in 99.5% ethanol for 10 min, then epitope retrieval was performed by adding the CINtecPLUS retrieval solution and incubating the specimen at 99 °C for 10 min. The cells were stained using the CINtecPLUS primary antibody for 30 min. Then, they were treated sequentially with secondary antibodies against Ki67 (Alexa Fluor 488, ab150105, Thermo Fisher Scientific, Tokyo, Japan), p16 (Alexa Fluor 594, ab150076, Thermo Fisher Scientific), and Hoechst (33342, Dojindo, Tokyo, Japan), followed by blocking with 1% bovine serum albumin in phosphate buffered saline (PBS) for 30 min. The stained cells were stored in PBS at 4 °C. Before their use, the fixed cells were resuspended in 100 µL of 3.5% Pluronic F-68 (PF-68, Gibco, Tokyo, Japan) to induce negative dielectrophoresis (nDEP).

### Cell trapping

Fixed cells loaded onto the access port of the device were delivered to the microfluidic channel by pulling out an airtight glass syringe using a syringe pump. The flow rate was set at 2 µL/min. The cells were trapped in an electroactive field via the application of a potential to an ITO electrode (10 V and 10 MHz). Prior to their use, the fixed cells were resuspended in low-resistance buffer (3.5% nonionic surfactant and Pluronic F-68 [PF-68, Gibco, Tokyo, Japan] dissolved in MilliQ water) to induce nDEP.

### Determining the number of stained cells

The cells were analyzed and counted using an automatic counting application, the BZ-X800 Analyzer's Hybrid Cell Count application (KEYENCE, Osaka, Japan), available within the fluorescence microscopy software. The number of cells stained with Hoechst was measured and used as the total number of cells within the field of view. The number of cells stained with Hoechst, Ki67, and p16 were measured and designated as the number of positive cells by multiple gynecologists; this facilitated the unbiased assessments of cell counting. Then, the positive cell rate was calculated as the number of positive cells divided by the total number of cells and multiplied by 100%. The median value of multiple locations was used in the final analysis.

### Immunohistochemistry

The representative cervical intraepithelial neoplasms (CIN) lesions were selected according to routine procedures. Immunohistochemistry with p16 and Ki67 was performed on formalin-fixed, paraffin-embedded tissue using an anti-p16 antibody (E6H4, Roche, Tokyo, Japan) and an anti-Ki67 antibody (MIB-1, Agilent Technologies International Japan, Tokyo, Japan). All the procedures were performed using BOND polymer refine detection systems (Leica biosystems, Tokyo, Japan) and the samples were evaluated by three independent pathologists as a routine pathological diagnosis.

### Statistical analysis

The study data collected were analyzed using by JMP Pro 14.0.0 software [SAS Institute Japan, Tokyo, Japan] using Mann–Whitney test with the level of significance set at *P* < 0.05.

## Data Availability

The data that support the findings of this study are available on request from the corresponding author on reasonable request.
